# Evaluating the sustainability of non-communicable diseases programs in Malaysia

**DOI:** 10.1186/s12889-022-13891-6

**Published:** 2022-08-01

**Authors:** Selvanaayagam Shanmuganathan, Feisul Idzwan Mustapha, Andrew Wilson

**Affiliations:** 1 Menzies Centre for Health Policy and Economics, School of Public Health, Faculty of Medicine and Health, Charles Perkins Centre (D17), The University of Sydney, Sydney, NSW 2006 Australia; 2 Ministry of Health, Putrajaya, Malaysia; 3 Disease Control Division, Ministry of Health, Putrajaya, Malaysia

**Keywords:** Program evaluation, Program Sustainability, Leadership, Chronic disease, Non-communicable Diseases

## Abstract

**Background:**

The substantial rise in non-communicable diseases (NCDs) over the last two decades poses a major concern to the healthcare services in Malaysia. This study aimed to evaluate the sustainability of the current NCDs programs and identify the challenges and factors impeding the sustainability of the NCDs program implemented under the National Strategic Plan.

**Methods:**

This study applied the mixed-method approach using the Program Sustainability Assessment Tool (PSAT) to assess the eight domains for program sustainability combined with 5 open-ended questions. The survey was administered to key leaders from the district health offices in Malaysia. The mean score for each sustainability domain and the overall mean sustainability score were determined. Descriptive statistics and thematic analysis were conducted using Statistical Package for the Social Sciences (SPSS) version 25 and NVivo version 12, respectively.

**Results:**

A total of 80 key leaders responded to the survey. Overall seven domains scored an average of ≥ 4 with an overall mean sustainability score of 4.2. The highest domain mean scores were 4.5 (communications) and 4.4 (organizational capacity). The lowest mean score domain was 3.8 (funding stability). The open-ended responses revealed challenges faced by department heads, including implementation difficulties, factors impeding the planning of the NCDs program for sustainability, lack of financial resources, lack of human resources, and support for staff training which are largely consistent with the scores of each domain.

**Conclusion:**

The sustainability factors affecting the NCDs program in Malaysia are qualitatively similar to other countries. For greater sustainability capacity, we should work towards strong leadership, strengthening funding stability, and incorporating evidence-based public health strategies in the implementation of the NCDs program.

## Background

Chronic diseases are well recognized as the major health challenge in developed and many emerging economies. Chronic disease management programs (CDMPs) involve planned organization of care aiming to help patients better self-manage their health, reduce risk factors, facilitate more consistent and coordinated clinical care, and reduce associated disease risks. Such programs include health assessments, action plans, patient education, and health behaviour tracking, with ongoing support [1, 2]. CDMPs have been shown to deliver improved health outcomes but typically need time to reach a certain level of maturity to allow health benefits to accrue. Maintaining and sustaining CDMPs over longer periods of time to achieve these benefits is challenging and there is a need to better understand what factors can promote long-term program sustainability.

Over time, a CDMP ideally must sustain activities described as ‘sustainability outcomes’ such as community-level partnerships, organizational practices, benefits to clients, and the salience of the program’s core issue [3]. There is a need to maintain a coordinated approach to chronic disease management from individual care and program efficiency perspectives. The overriding challenge is ensuring the continuation of effective health programs beyond the implementation stage as there is limited research on what happens to programs once they have been implemented [4].

Sustainability is defined as “the existence of structures and processes that allow a program to leverage resources to effectively implement and maintain evidence-based policies and activities” [5]. The sustainability concept also includes strong organizational infrastructure and leadership. Program sustainability is complex and factors that are necessary to ensure sustainability for different interventions with diverse contexts have not been fully untangled [6, 7]. As a result, Luke *et al* developed a Program Sustainability Framework to assess public health program capacity for sustainability, which includes eight domains (organizational capacity, program adaptation, program evaluation, communications, strategic planning, funding stability, environmental support, and partnerships) [5, 7, 8].

In Malaysia, most patients with NCDs are managed at the primary healthcare level. This activity is one of the main workload and organizational challenges faced by all primary healthcare providers. Malaysia has a dual primary health care system which consists of both public and private sectors with the public sector as the mainstream health provider. The Malaysian public primary healthcare service has developed significantly since the 1950s by establishing primary health clinics (PHCs). The PHCs provide easy access to health care for the community, with each clinic serving a population of approximately 15,000–20,000 population. The primary healthcare system in Malaysia provides basic or general healthcare focusing on the point at which a patient first seeks assistance from the medical care system. The referral system connects the primary healthcare facilities with hospitals (at the district and state level) and specialist centres [9]. Primary healthcare gradually evolved from providing maternal and child care services to acute care of infectious diseases and minor ailments. The increasing burden of NCDs required the need for and focused on chronic care, and this challenged the existing system with available resources designed to cater to acute care.

The primary healthcare system in Malaysia adopted the ‘Reviewed Approach of Primary Healthcare’ (REAP-WISE) to service delivery in 2007 [10]. The REAP-WISE framework represents the various component of health services provided at primary care clinics, such as Wellness (health promotion, screening, and identification of risk factors; Illness intervention and treatment); Support services (rehabilitation and follow-up care; and Emergency services. Multi-disciplinary and skilled primary healthcare teams were introduced to clinics that adapted to a more integrated approach [11]. The current primary healthcare clinics mainly include family medicine specialists, general medical practitioners, physiotherapists, occupational therapists, nurses, assistant medical officers, nutritionists, and dieticians.

In response to the rise in NCDs, the Ministry of Health, Malaysia implemented “The National Strategic Plan for Non-Communicable Diseases (NSP-NCD) 2010–2014,” followed by the 10-year National Strategic Plan for Non-Communicable Diseases (NSP-NCD) 2016–2025 to address the burden of NCDs at national and state level [12]. The initial NSP-NCD 2010–2014 was developed based on the mandates of the World Health Organization (WHO), particularly with reference to the “2008–2013 Action Plan for the Global Strategy for the Prevention and Control of NCDs” and the “Western Pacific Regional Action Plan for NCDs.” The current focus of NSP-NCD 2016–2025 is on three types of NCDs (cardiovascular diseases, diabetes mellitus, and cancer) and four shared NCD risk factors (tobacco use, unhealthy diet, physical inactivity, and harmful use of alcohol) [13].

This study aimed to (i) evaluate the sustainability of the NCDs program implemented under the NSP-NCD in Malaysia and (ii) identify challenges and factors impeding the sustainability of the NCDs program implemented in the primary healthcare settings within the Ministry of Health (MOH). The paper covers the results of a survey of key managers in district health offices across Malaysia.

## Methods

### Setting and study design

A survey of leaders in district health offices (DHO) was conducted using a questionnaire compromising the Program Sustainability Assessment Tool (PSAT) (version 2) and five open-ended questions. Respondents were recruited from January to April 2019. The translation was not necessary as all respondents were fluent in English. Ethical approval for this study was obtained from the Medical Research and Ethics Committee (MREC)-NMRR-18–2542-44,097 (IIR), Ministry of Health, Malaysia.

### Instrument

The PSAT is a 40-item multiple-choice instrument, that assesses the program’s sustainability capacity in 8 domains (organizational capacity, program adaptation, program evaluation, communications, strategic planning, funding stability, environmental support, and partnerships). The responses to each item are recorded on a 7-point Likert scale from “to little or no extent” [1] to “to a great extent” [7]. The mean score for each of the eight sustainability domains and the overall mean sustainability score was calculated. The overall sustainability score can range between 1 and 7, with the higher score indicating greater strength in the domain [5].

### Data analysis

Descriptive statistics were used to summarize the data. Data analysis was performed using Statistical Package for the Social Sciences (SPSS) version 25. NVivo was used to manage the qualitative data. Thematic analysis was conducted for the five open-ended questions: (1) Who are your champions or advocates? In what ways do they advocate for the program (or have they advocated, or you hope they will do)? (2) What organizations or individuals are invested in the success of the program? Why? (3) What are the strengths and weaknesses you see in terms of the organizational capacity to maintain the existing NCD program? (4) What is the current funding situation? (5) What have you learned (or done) about creating sustainable care coordination programs or improvements? Two researchers coded open-ended questions independently and discussed them to ensure convergence and divergence of the coding scheme. Subsequently, themes were identified for the eight domains of the sustainability framework.

## Results

From the 114 DHOs across Malaysia, a total of 80 respondents agreed to participate in this study. The overall response rate was 70.2%. The median age was 47 years (range 36 − 60) and the majority of respondents were female (63%) (Table 1). More than half of the respondents (56%) were in service for more than 20 years and sixty percent were based in an urban district health office.

**Table 1:** Participants’ socio-demographic (*n* = 80)

Characteristics	n (%)^a^
Age (years), median (range)	47 (36–60)
Age > 45	50 (63)
Age ≤ 45	30 (37)
Sex	
Female	50 (63)
Male	30 (37)
Length of service (years), median (range)	21.5 (11–35)
≤ 20	35 (44)
> 20	45 (56)
Locality of local health district	
Urban	48 (60)
Rural	32 (40)

^a^Data are presented in n (%) unless otherwise stated

**Fig. 1:**
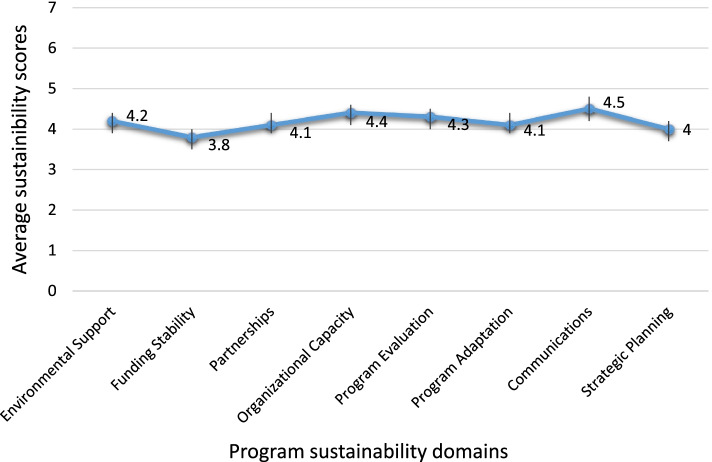
Distributions of Mean Sustainability Scores for 80 districts with sustainability domains. Each line represent the minimum and maximum values for each domain

The overall mean sustainability capacity score and standard deviation across sites was (4.2, SD: 1.0). The mean score for 8 domains were Communications (4.5, SD: 1.2), Organizational capacity (4.4, SD: 1.1), Program evaluation (4.3, SD: 1.1), Environmental support (4.2, SD: 1.1), Program adaptation (4.1, SD: 1.2), Partnerships (4.1, SD: 1.2), Strategic planning (4.0, SD: 1.3) and Funding stability (3.8, SD: 1.1) (Fig. 1). Responses from the open-ended questions which provided insights into the domain ratings are presented below in descending order of average domain score.

Of relevance to the domain of Communications, responses clearly supported communications as a key to the implementation of the NCDs program. While there was open communication between leaders and staff, it primarily followed organizational management lines, a top-down approach at all levels of the organization. It was also reported that the success of programs in certain districts relied on good relationships and collaboration between staff and community leaders. Good communication from NGOs to advocate the program together with DHO was also important. Some respondents reported a need for support from the government to improve communication with other government agencies for the successful implementation and sustainability of the NCDs program.

Of relevance to the domain of Organizational Capacity, respondents reported various strengths in promoting the implementation of NCD activities, good integration of clinical work, and NCDs program. There are ongoing challenges at the district level of multiple competing priorities in addressing the NCDs program. Other commonly cited challenges were resource-related, such as lack of office space, adequate staff numbers, staff training, and generally limited resources and assets to run the NCDs program. Some DHO leaders pointed to the importance of providing their staff with opportunities for growth and involving staff at all levels in the NCDs program’s decision-making process.

Of relevance to the domain of Program Evaluation, the NSP-NCDs program evaluation process is undertaken yearly. Findings are used to strengthen the NCDs program and its sustainability by identifying issues with the program and continuously improving the program. There were suggestions on strengthening the evaluation process with sufficient resource allocation, seeking regular feedback from operational staff (bottom-up approach) and other stakeholders to improve the program. Some mentioned that lack of access to health record data and inadequate process evaluation limited the effectiveness of evaluation on sustainability.

Of relevance to the domain of Environmental Support, open responses indicated it is a crucial and underlying element to implementing the NCDs program and its sustainability. The continuous economic support and internal politics (stability within the organizational structure and climate) were crucial in implementing and sustaining the NCDs program at the district level. Some respondents mentioned good political support resulted in advocacy of the NCDs program within the community and strong participation from the community in the NCDs program. There is a need for external support from the community to gain broad public support for the initiative in sustaining the NCDs program.

Of relevance to the domain of Program Adaptation, comments reinforced that it was critical in the success and sustainability of the NCDs program. Adaptability was seen as important for effective collaboration with key partners, adapting current workflow to accommodate the NCDs program, engagement of clinicians in health promotion activities apart from clinical management of NCDs, and leveraging resources. Currently, the nurses and medical assistants are tasked to carry out the screening, monitoring, and health promotion activities. Respondents emphasised the need for understanding local issues and challenges prior to implementing new programs.

Of relevance to the domain of Partnerships, comments reinforced that these were crucial in implementing and sustaining the NCDs program. The core process for maintaining the programs was building strategic partnership management and creating coalitions among the community and other agencies. The DHO leaders recognised the importance of sustaining partnership, and building trust with the community was vital to maintaining activities for the community when overcoming challenges of limited resources and funding. Engaging community and religious leaders were a critical factor in program acceptability within the community.

Of relevance to the domain of Strategic Planning, the respondents described the need for more effort and progress in planning to ensure implemented NCDs program would last. Some DHOs encountered implementation barriers in terms of funding and suggested a focus on sustainability planning of all programs prior to the rollout of new programs. In some instance, a lack of clear understanding of the roles and responsibilities among stakeholders impact the outcome of the NCDs program. There is a concern about the implementation of new programs with no integration or continuity with existing programs. Some reported that involving staff from districts in aspects of strategic planning was important to ensure success of the programs.

Of relevance to the domain of Funding Stability, responses reinforced its importance in sustaining the NCDs program and providing greater flexibility of funding programs at the district level. Issues raised included strengthening funding by cost-sharing, support from external resources, and being flexible about which program components are supported. In certain districts, limited funds impacted the implementation and sustainability of NCDs programs.

## Discussion

The sustainability of the NCDs programs is vital to the public health effort and a better understanding of the strengths and challenges of the existing programs will help guide the federal and state governments in policymaking decisions. As part of a broader evaluation of the NCDs program at the district level across all states in Malaysia, we used the PSAT to evaluate sustainability capacity in 8 domains with open-ended questions. The PSAT tool was designed to be easy to use for a wide variety of community and public health programs [5, 8]. High scores indicate that a domain is considered strong for program sustainability while lower scores indicate areas of concern. In this study, the sustainability capacity domain scores were fairly consistent across sites and in comparison to other studies using PSAT as a tool [14, 15].

The domains of communication and organizational capacity were rated highly at all sites. Funding stability is a known barrier or challenge described in the literature and was the most frequently reported barrier to sustainability in 48 projects with short-term foundation funding [16]. Communication and organizational capacity with good leadership support for building capacity, program evaluation, program adaptation, partnership, strategic planning, environmental support, and funding stability are important factors in sustaining the NCDs program. The study found funding stability score was relatively low as compared to other domains. The growing burden of NCDs requires a regular and gradual increase in funding for screening, equipment, medications, laboratory tests, training, and resource allocations. In Malaysia, where the government funds approximately 70% of the patients’ treatment cost, most patients seek treatment in public health care facilities [17].

NCDs management requires long-term care; accessibility and affordability are regarded as two critical factors for NCD treatment. Hence, public primary healthcare became the leading choice by the rural and urban communities. NCDs can be prevented by managing risk factors and early detection of the disease. The main challenge is addressing the issue and creating awareness among the public to visit health care facilities at the early stage of NCDs where they are free from any symptoms [11]. Initiatives were undertaken to create more capacity within the community to implement community-based interventions with the collaboration and engagement of community health care volunteers and community members of Health Clinic Advisory panels to address and provide a local community perspective on local health issues. This approach is essential and valued by the MOH, given the effective implementation of health interventions related to chronic conditions are unlikely to be successful without community support [11].

Improving communication within the various levels of MOH is vital in program sustainability. Increased top-down communication would help to build an internal agency culture that would be more resilient to external conflicts, such as funding instability or complicated political environments. The communication process within the organization should reflect a process of discussion, engagement, clarification, negotiation, and perspective-taking, rather than solely as information exchange or directive. A review of administrative and management to improve local public health recognised participatory decision-making, involving communication with employees to get their input as an effective way to create a conducive working environment [18], essential for program sustainability. Efforts from the upper management (federal level) within the MOH to improve internal communication might include incorporating participatory decision-making.

The study found that the role of leadership in communication was essential and mentioned consistently, that constant communication of information and coordination of the program is vital for success in sustainability [19]. The absence of clarity and consistent communication among leaders of an organization, even across multiple levels within the organization, can influence the implementation and impact the program’s success [20]. Leadership and organizational support are key features because leaders can ensure program sustainability, providing necessary allocation of resources (human and material), and indirectly via encouragement, support, and mentorship [21]. There are similar findings from other health fields with reports that “leadership is critical to building organizational readiness for change” [20, 22]. We found that leadership was important in most of the sustainability domains and that some districts struggled with implementing and sustaining programs partly due to lack of support from leadership at upper management level within the MOH. Leaders at a district level maybe responsible for many programs including NCDs. Their priorities may, intentionally or unintentionally, affect what type of programs are implemented and how they are measured or evaluated [15, 23]. Flexibility to manage funds by leaders at the district level is important for the overall sustainability of the program [22]. There is a need for collective leadership at all levels within the MOH for successful program implementation and sustainability.

There are many factors that affect the NCDs program’s capacity for sustainability. Funding is crucial for the implementation and sustaining of the NCDs program, and factors such as organizational support, partnerships, and communications can aid in sustaining the NCDs program. The PSAT and planning process provide public health programs and their partners with a reliable method for rating their programs’ capacity for sustainability. This method is unique in that programs can identify their areas of strength and weakness across a range of sustainability factors and then make informed decisions about where to concentrate efforts. The study focused on leaders of the DHOs, which may limit the generalizability of the findings within the organization. The qualitative results provide additional support for the association between the domains and the findings from the empirical literature on sustainability [24], support the interactive relationships that influence the sustainability of a program, and provide insight into how respondents interpreted the tool items for each domain [25]. This additional information supports the validity of scale items, as described by the scale developers [5]. These findings highlight the importance of examining PSAT scores by item and domain and overall average to gain a complete understanding of the NCDs program’s strengths and opportunities for improvement. The findings may be most relevant to interventions that require coordination among multiple agencies and approaches to addressing the burden of NCDs that influences sustainability depending on the type of intervention [26].

The NCDs program is being evaluated by MOH regularly, and the study highlighted the factors and challenges of sustaining the NCDs program at the district level. The findings from this study may help improve the evidence-based approach for program sustainability in DHO settings. The evaluation was by the district leaders of the existing NCDs program, with the qualitative findings revealing the success and challenges faced at the ground level and aid policymakers at the federal level in the future implementation of policies and planning of the NCDs program.

Since this is the first study evaluating the sustainability of the non-communicable disease program at the district level in Malaysia, future studies could adapt the PSAT to assess long-term program sustainment. Investments in leadership, improvement in communication from the top of the organization, and greater funding flexibility may enhance the sustainability of evidence-based public health non-communicable diseases programs.

## Conclusion

The findings from this study revealed that in the Malaysian context, a key area perceived as needing improvement for NCD program sustainability is the need for greater security for continued funding to support staff. Organizational capacity, program evaluation, program adaptation, communication, environmental support, strategic planning, and funding stability appeared to be lesser issues than whether a DHO can sustain programming. Increased top-down and bottom-up communication would help build an internal culture for better resilience to external conflicts such as uncertain political environments and funding instability. This study also demonstrated the usefulness of the PSAT for guiding a mixed-methods evaluation of sustainability capacity for the NCDs program. Findings from this study may help improve the sustainability of the NCDs program in DHO settings. Program managers can use the PSAT to assess the program design and implementation process to identify links between these and long-term program sustainment.

## Data Availability

Data is available on request from the corresponding author.

## Abbreviations

NCDs: Non communicable diseases
DHO: District Health Office
PSAT: Program Sustainability Assessment Tool
CDMPs: Chronic disease management programs
WHO: World Health organization
NSP-NCD: National Strategic Plan for Non-Communicable Diseases
MREC: Medical Research and Ethics Committee, MOH: Ministry of Health
